# Degradation of Methylene Blue Using Biologically Synthesized Silver Nanoparticles

**DOI:** 10.1155/2014/742346

**Published:** 2014-03-19

**Authors:** M. Vanaja, K. Paulkumar, M. Baburaja, S. Rajeshkumar, G. Gnanajobitha, C. Malarkodi, M. Sivakavinesan, G. Annadurai

**Affiliations:** Environmental Nanotechnology Division, Sri Paramakalyani Centre for Environmental Sciences, Manonmaniam Sundaranar University, Alwarkurichi, Tamil Nadu 627412, India

## Abstract

Nowadays plant mediated synthesis of nanoparticles has great interest and achievement due to its eco-benign and low time consuming properties. In this study silver nanoparticles were successfully synthesized by using *Morinda tinctoria* leaf extract under different pH. The aqueous leaf extract was added to silver nitrate solution; the color of the reaction medium was changed from pale yellow to brown and that indicates reduction of silver ions to silver nanoparticles. Thus synthesized silver nanoparticles were characterized by UV-Vis spectrophotometer. Dispersity and morphology was characterized by scanning electron microscope (SEM); crystalline nature and purity of synthesized silver nanoparticles were revealed by X-ray diffraction (XRD) and energy dispersive X-ray spectroscopy (EDX). FTIR spectrum was examined to identify the effective functional molecules responsible for the reduction and stabilization of silver nanoparticles synthesized by leaf extract. The photocatalytic activity of the synthesized silver nanoparticles was examined by degradation of methylene blue under sunlight irradiation. Green synthesized silver nanoparticles were effectively degrading the dye nearly 95% at 72 h of exposure time.

## 1. Introduction

Nanotechnology deals with the synthesis of nanoparticles with controlled size, shape, and dispersity of materials at the nanometer scale length [1] and their potential use for human well-being. Nanometer sized materials have a high surface area; and a high fraction of surface atoms [2] have been studied because of their exclusive properties such as optic, electronic, and catalytic [3–7]. Among all nanoparticles noble metal nanoparticles have enormous applications in diverse areas such as bioimaging, sensor, diagnosis, and novel therapeutic in biomedical field [8]. Metallic silver and silver nanoparticles were recently applied as antimicrobial agents in various products such as cosmetics [9], animal feed [10], coating of catheters [11], wound dressing [12], and water purification [13] with a minimal risk of toxicity in humans.

Nowadays the biological systems were eagerly used for nanoscale material synthesis and assembly is an alternative method of physical and chemical process. Green approach of nanoparticles synthesis by biological entities has been gaining great advantages which are environmental benign, less toxic, and time consuming; and also it is a single step process [14]. Currently, plant and plant derived materials are used for nanoparticles synthesis which is more compatible than the microbe-mediated nanoparticles synthesis process because they eliminate the culture maintenance and are easy to handle [15]. Nanoparticles synthesis by medicinal plants shows more benefit; they may enhance the antibacterial activity of silver nanoparticles, because the medicinally valuable active biomolecule present in the plants may bind on the surface of the nanoparticles and reduce the silver ions to silver nanoparticles.

*Morinda tinctoria* commonly known as Aal or Indian Mulberry is a species of flowering plant in the family Rubiaceae. The whole body of this plant has many medicinal properties. Leaves are used for curing ulceration, digestion, dyspepsia, diarrhea, stomatitis, wound, and fever. The leaf juice is used as a local application. The root is used to cure inflammation and boils [16, 17]. The unripe fruit is used to cure rheumatism [18–21]. In this study, we successfully reported the biosynthesis of silver nanoparticles using* M. tinctoria* leaf extract. Synthesized silver nanoparticles were applied to dye degradation under sunlight irradiation.

## 2. Materials and Methods

### 2.1. Preparation of Green Reducing Agent

Leaves of* Morinda tinctoria* were collected from the Sri Paramakalyani Centre for Environmental Sciences campus, Manonmaniam Sundaranar University, Alwarkurichi, Tamil Nadu, India. 10 g wet weight of fresh leaves was cut into fine pieces and washed with distilled water and boiled with 100 mL of double distilled water for 10 min at 60°C. Boiled mixture was filtered through Whatman No. 1 filter paper and collects the supernatant of leaf extract and stored at 4°C for further nanoparticles synthesis process.

### 2.2. Phytosynthesis of Silver Nanoparticles

Aqueous solution of silver nitrate was prepared using double distilled water at a concentration of 1 mM. Silver nitrate was purchased from HiMedia, Mumbai. 10 mL of freshly prepared leaf extract was added to 90 mL of aqueous solution of silver nitrate and kept at room temperature for the reduction of silver ions to silver nanoparticles. Nanoparticles formation was visually identified by color change and followed the UV-Vis spectrum analysis. The pH of leaf extract was altered to study its effects on synthesis of silver nanoparticle. The various pH (4.6, 5.6, 6.6, 7.6, and 8.6) of the 10 mL of leaf extract were added into 90 mL of 1 mM silver nitrate solution. The pH was adjusted by using 0.1 N NaOH and 0.1 N HCl. Formation of silver nanoparticles was measured by UV-Vis spectrophotometer at different wavelengths.

### 2.3. Characterization of Phytosynthesized Silver Nanoparticles

The reduction of silver ions was monitored by measuring double beam UV-Vis spectra of the reaction medium at different wavelenthgs from 360 to 700 nm at different functional time (PerkinElmer, Singapore). The silver nanoparticle solution thus obtained was purified by repeated centrifugation at 7000 rpm for 15 min and dried at 100°C. Crystalline nature of the nanoparticles was analyzed by XRD at 2*θ* ranges from 20 to 80°C (Philips PW 1830). The morphology and size of the silver nanoparticles were found by Scanning Electron Microscope (Philips CM-200). Elemental analysis of silver was carried out by EDX (Philips XL-30). Functional biomolecules associated with silver nanoparticles were confirmed by FTIR, which is involved in the reduction of silver ions into silver nanoparticles. The FTIR spectrum was obtained on a Shimadzu instrument with the sample as KBr pellet in the wavenumber region of 500–4,000 cm^−1^.

### 2.4. Photocatalytic Degradation of Dye

Typically 10 mg of methylene blue dye was added to 1000 mL of double distilled water used as stock solution. About 10 mg of biosynthesized silver nanoparticles was added to 100 mL of methylene blue dye solution. A control was also maintained without addition of silver nanoparticles. Before exposing to irradiation, the reaction suspension was well mixed by being magnetically stirred for 30 min to clearly make the equilibrium of the working solution. Afterwards, the dispersion was put under the sunlight and monitored from morning to evening sunset. At specific time intervals, aliquots of 2-3 mL suspension were filtered and used to evaluate the photocatalytic degradation of dye. The absorbance spectrum of the supernatant was subsequently measured using UV-Vis spectrophotometer at the different wavelength. Concentration of dye during degradation was calculated by the absorbance value at 660 nm.

Percentage of dye degradation was estimated by the following formula:

(1) $$\begin{array}{c} \%\text{Decolourization}=100\times \frac{\left(C_{0}-C\right)}{C_{0}}, \end{array}$$

where *C*_0_ is the initial concentration of dye solution and *C* is the concentration of dye solution after photocatalytic degradation.

## 3. Results and Discussion

### 3.1. Optical Observation

**Figure 1:**
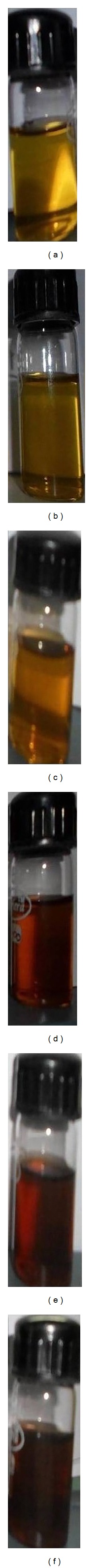
Visual identification of silver nanoparticles synthesized by* M. tinctoria* leaf extract at pH 8.6 as recorded at different functional time ((a) initial, (b) 10 min, (c) 30 min, (d) 1 h, (e) 2 h, and (f) 4 h). The formation of dark brown colour revealed the formation of silver nanoparticles in the reaction mixture.

Initially, while adding the leaf extract of* Morinda tinctoria* to the silver ion solution, the color of the solution was turned into yellowish brown which indicates the formation of silver nanoparticles [22] (Figure 1). The formation of color occurred due to the excitation of surface Plasmon resonance of the silver nanoparticles [23]. The result obtained in this investigation is very interesting in terms of identification of potential plants for synthesizing the silver nanoparticles [24]. Similarly, Govindaraju et al. [25] observed the color change to brownish yellow while synthesizing silver nanoparticles using the leaf extract of* Solanum torvum*. Rao and Savithramma [26] also reported that the* Svensonia hyderabadensis* solution of the silver ion complex started to change the color from yellow to dark brown due to the reduction of silver ions. Chen et al. [27] reported the intensity of the color development in the reaction mixture of different plants such as in* Helianthus annuus, Basella alba,* and* Saccharum officinarum*.

Role of pH in the synthesis and nature of silver nanoparticles was investigated by changing the experiment pH which was characterized by color change of reaction mixture and UV-Vis spectrophotometer. pH plays an important role in the synthesis and controlling size and shape of nanoparticles. The colour and the intensity peaks of nanoparticles were pH dependent. At pH 4.6, no color change occurred. It indicates acidic pH suppresses the nanoparticles synthesis. At pH 5.6, the yellow colour was formed at 10 min incubation and turned into brown colour at 30 min which indicates formation of silver nanoparticles. It was similarly observed in the following pH 6.6. At 1 h time of incubation both show dark blackish brown color with precipitation which indicates completion of nanoparticles synthesis. At high pH 7.6 and 8.6, the brown colour was maintained for several weeks without precipitation which indicates stabilized synthesized nanoparticles (Figure 1). Under the acidic conditions such biomolecules are likely to be inactivated so that the nanoparticles synthesis could not occur at pH 4.6. The differences in the arising of color over the various pH could be due to the presence of various dissociated functional groups on the leaf extract that are actively involved in the synthesis process [28]. At higher pH, however, more number of small sized nanoparticles was synthesized due to the availability of large number of functional groups for silver binding [29]. Sathishkumar et al. [29] reported that higher pH influences the formation of more of spherical shape rather than ellipsoidal silver nanoparticles was synthesized by using* Cinnamon zeylanicum* bark extract. Interestingly, even high pH 8.6 was also found to be efficient in producing nanoparticles, but they agglomerated within few days.

### 3.2. UV-Vis Spectrophotometer Analysis

**Figure 2:**
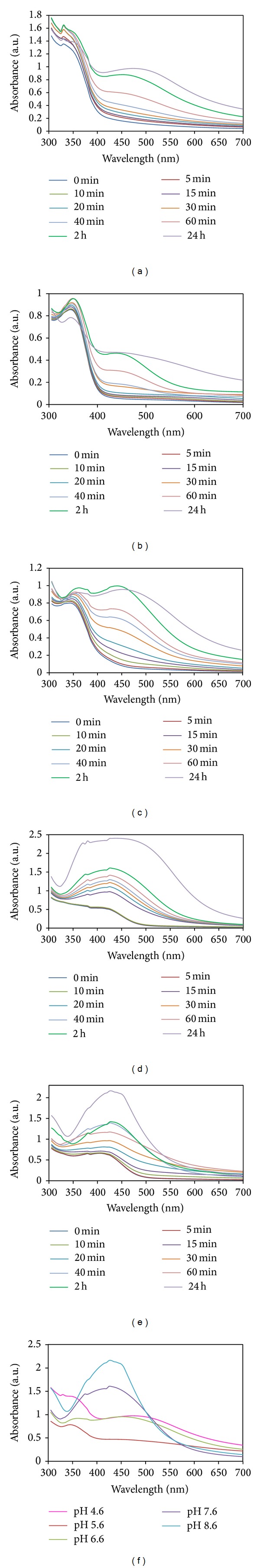
UV-vis spectrum of silver nanoparticle synthesized by leaf extract of* M. tinctoria* recorded at different pH ((a) pH 4.6 (b) pH 5.6 (c) pH 6.6 (d) pH 7.6 (e) pH 8.6) and different incubation period (0–24 h), (f) Comparison of synthesized silver nanoparticle at different pH from 4.6 to 8.6 and the incubation time is 24 h.

pH plays an important role in the nanoparticles synthesis; this factor induces the reactivity of leaf extract with silver ions.  Figure 2 shows the effect of pH on the nanoparticles synthesis. Acidic pH 4.6 and 5.6 show the peak between 320 and 350 nm initially, and then these were maintained till 1 h of incubation time. After 1 hr, another band appeared at 450 nm with broadened nature indicating the formation of larger sized nanoparticles. After 24 h, the peak was changed into 470 nm with high agglomeration due to the lack of stabilizing agent (Figures 2(a) and 2(b)). At pH 6.6 initially the peak occurred at 340 nm, and the second band was formed at 420–430 nm indicating the synthesis of silver nanoparticles. After 24 h, the peak positions were changed into 360 and 470 nm due to the aggregation of nanoparticles (Figure 2(c)). The lower absorbance was observed in the acidic pH due to suppression of nanoparticles synthesis. In the alkaline pH 7.6 and 8.6, nanoparticles synthesis was high by observing the absorbance (Figures 2(d) and 2(e)).

In the alkaline pH, the SPR band was positioned at 380 nm and 420 nm by forming the narrow peak indicating particles are in small size. High stabilized nanoparticle was synthesized at the alkaline pH (Figure 2(f)). With increasing the pH of the reaction the optical absorbance was increased and also small sized nanoparticles were synthesized very quickly. In the low pH the nanoparticles were agglomerated and formed large sized nanoparticles [30]. Size, shape and distribution of nanoparticles were depending on the formation of one or more surface Plasmon resonance (SPR) bands. Formation of a single SPR band at short wavelengths revealed the presence of small sized spherical nanoparticles in the reaction mixture, whereas two or more SPR bands were shown at larger wavelengths indicates presence of large anisotropic nanoparticles [31]. Therefore, the narrow peak at 420 nm is likely shown due to formation of small spherical silver nanoparticles. Similarly the two bands for silver nanoparticles were early reported by Kumar et al. [31]. Silver nanoparticles were highly synthesized with small size in the alkaline pH due to the availability of large amount of positive functional groups in the leaf extract that allows silver ions to get more binding sites [32]. Similarly, Dwivedi and Gopal [33] reported synthesized nanoparticles are stable under a wide pH range and also they elaborated that lower pH 2 shows lower and broader absorbance as compared to the pH 4 onwards which can be due to the formation of larger nanoparticles.

### 3.3. Scanning Electron Microscope

**Figure 3:**
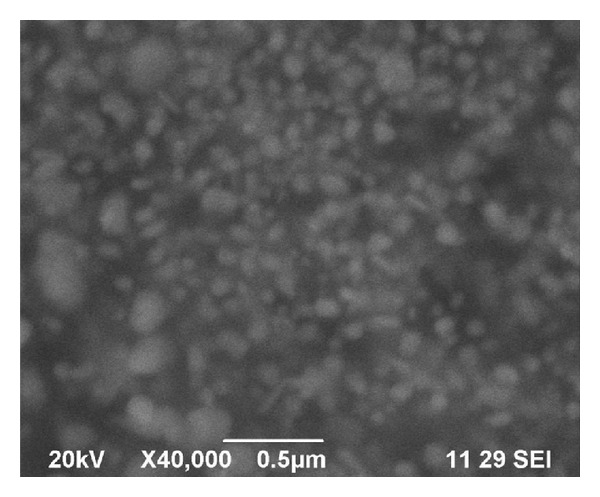
SEM images of silver nanoparticles synthesized from* M. tinctoria* leaf extract show highly agglomerated spherical shape.

SEM image shows the size and shape of the biosynthesized silver nanoparticles using* M. tinctoria* leaf extract. Size of the nanoparticles was observed at different magnifications. Spherical and rod shape of nanoparticles with high agglomeration was noted with the size range from 79 to 96 nm. In this SEM image, some of the nanoparticles show large size due to the aggregation of small size of nanoparticles (Figure 3). Polydispersed nanoparticles were observed in SEM image and revealed the result of UV-Vis spectrophotometer. The surfaces of aggregated nanoparticles were shown to be rough. Similarly, aggregation of nanoparticles was earlier reported by Ramgopal et al. [34] by using the extract of soap nuts in the reduction of silver ions. Aggregation of nanoparticles took place due to the insufficiency of capping agent in the leaf extract to synthesis of nanoparticles.

### 3.4. XRD and EDX

**Figure 4:**
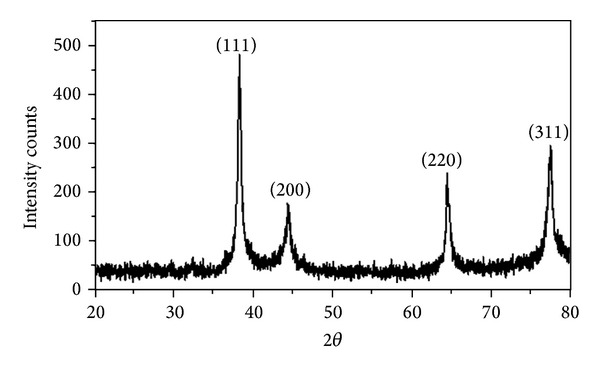
XRD spectrum of green synthesized silver nanoparticles.

Crystalline size and structure of the silver nanoparticles were carried out by XRD. The biosynthesized silver nanostructure by employing* M. tinctoria* leaf extract was further demonstrated and confirmed by the characteristic peaks observed in the XRD image (Figure 4). The four distinct diffraction peaks of the 2*θ* values of 38.26°C, 44.44°C, 64.58°C and 77.67°C can be assigned to the planes of (1 1 1), (2 0 0), (2 2 0), and (3 1 1), respectively, which indicates the silver nanoparticles are fcc and crystalline in nature (JCPDS file nos. 84-0713 and 04-0783). The broadening of Bragg's peaks indicates the formation of nanoparticles.

**Figure 5:**
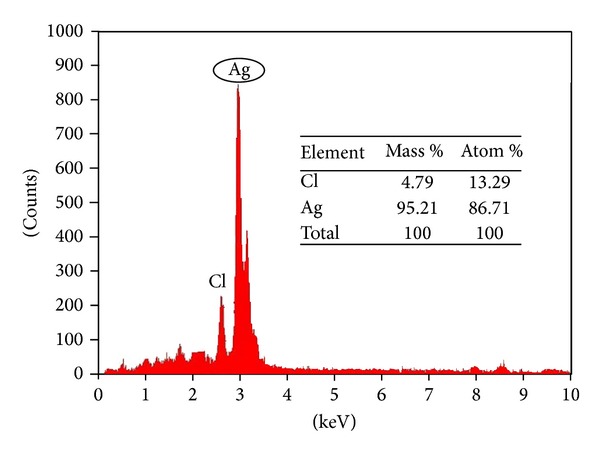
EDX spectrum of synthesized silver nanoparticles by* M. tinctoria* leaf extract.

Elemental analysis of silver was measured by EDX; EDX spectra reveal strong signals in the silver region of 3 keV and confirm the formation of nanosilver and its elemental nature. This signal was formed due to the excitation of surface Plasmon resonance of silver nanoparticles. Some of the weak signals from Cl were observed. These signals were found due to maybe the presence of impurity from the biological molecules of leaf extract (Figure 5).

### 3.5. FTIR

**Figure 6:**
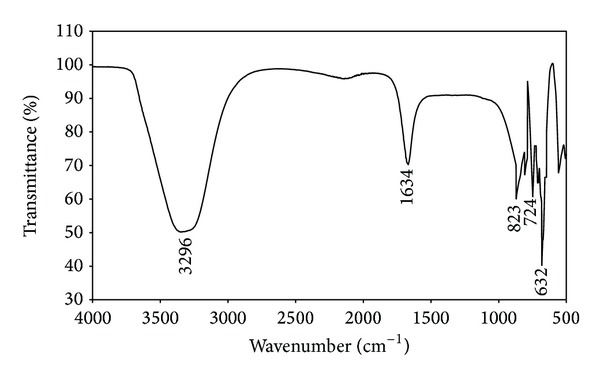
FTIR spectrum of aqueous leaf extract of* M. tinctoria*.

**Figure 7:**
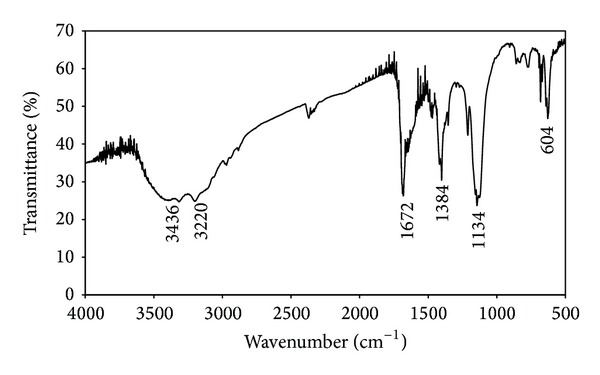
FTIR spectrum of green synthesized silver nanoparticles using* M. tinctoria* leaf extract.

FTIR measurements were carried out to identify the potential functional groups of the biomolecules in the leaf extract of* M. tinctoria* which are responsible for the reduction of the silver ions into silver nanoparticles (Figures 6 and 7).  Figure 6 shows a strong absorption peak at 3296 cm^−1^ which indicates presence of carboxylic groups. This functional group was modified in synthesized silver nanoparticles. The broad absorption band was observed between 3436 and 3220 cm^−1^ due to the O–H stretching and H- bonded alcohols and phenol groups (Figure 7). A weak band was observed at 1634 cm^−1^ corresponding to N–H bending primary amines. It was modified into 1672 cm^−1^ indicating presence of C=O stretching vibrations of carbonyls groups, respectively. New narrow bands were formed in the synthesized silver nanoparticles at 1384 cm^−1^ and 1134 cm^−1^ owing to the –NO_2_ aliphatic nitro groups and C–N stretching of aliphatic amines and C–O stretching of carboxylic groups. The two weak bands at 823 cm^−1^ and 724 cm^−1^ disappeared in the synthesized silver nanoparticles. A small peak was formed at 604 cm^−1^ due to the occurrence of alkyl halides (Figure 7). Moreover, the functional biomolecules are hydroxyl, carboxylic, phenol, and amine groups in* M. tinctoria* leaf extract involved in the reduction of silver ions which was confirmed by FTIR spectrum. Nagati et al. [35] reported that the aliphatic amine, aliphatic alkenes of alkaloids, and terpenoids bound on the surface of* Cajanus cajan* leaf extract mediated synthesized AgNPs.

### 3.6. Photocatalytic Degradation of Dye

#### 3.6.1. Visual Observation

**Figure 8:**
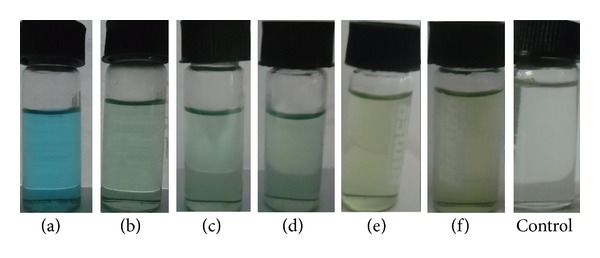
Visual observation of color change from blue to colorless indicates degradation of methylene blue dye at different time intervals ((a) initial, (b) 1 h, (c) 4 h, (d) 24 h, (e) 48 h, and (f) 72 h).

Photocatalytic degradation of methylene blue was carried out by using green synthesized silver nanoparticles under solar light. Dye degradation was initially identified by color change. Initially, the color of dye shows deep blue color changed into light blue after the 1 h of incubation with silver nanoparticles while exposed to solar light (Figure 8). Thereafter light blue was changed into light green. Finally, the degradation process was completed at 72 h and was identified by the change of reaction mixture color to colorless.

#### 3.6.2. UV-Vis Spectrophotometer

**Figure 9:**
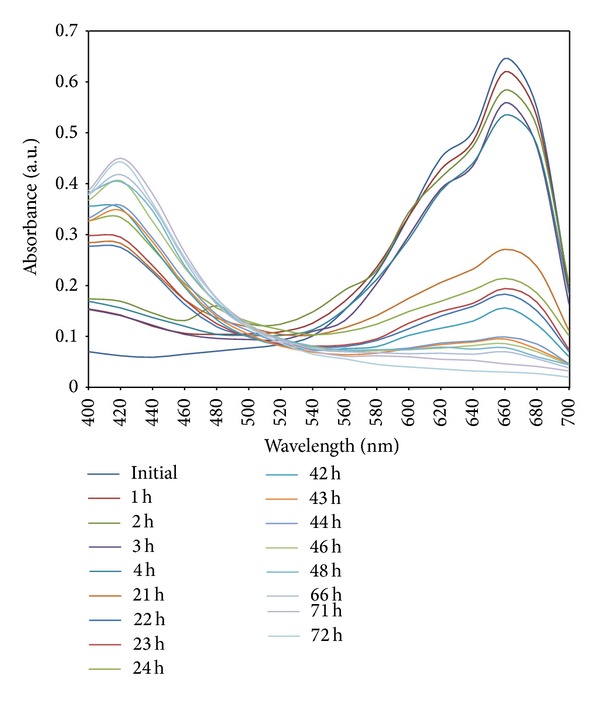
The absorption spectra of aqueous solution of methylene blue treated with 10 mg of synthesized silver nanoparticles using* M. tinctoria* at different time intervals.

**Figure 10:**
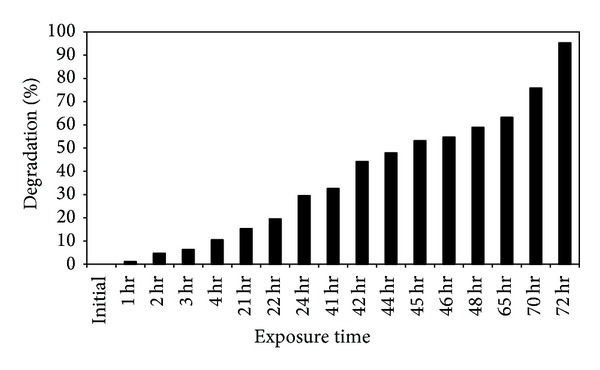
Percentage of dye degradation by 10 mg of synthesized silver nanoparticles at different functional time intervals.

Photocatalytic activity of silver nanoparticles on degradation of dye was demonstrated by using the dye methylene blue. The degradation of methylene blue was carried out in the presence of silver nanoparticles at different time in the visible region. The absorption spectrum showed the decreased peaks for methylene blue at different time intervals. Initially, the absorption peaks at 660 nm for methylene blue dye were decreased gradually with the increase of the exposure time and that indicates the photocatalytic degradation reaction of methylene blue. The absorption peak of methylene blue dye was decreased, and absorption band for silver nanoparticles was increased at 420 nm. The completion of the photocatalytic degradation of the dyes is known from the gradual decrease of the absorbance value of dye approaching the base line and increased peak for silver nanoparticles. While decreasing the concentration of dye, UV spectra show typical SPR band for silver nanoparticles at 22 h of exposure time (Figure 9). The percentage of degradation efficiency of silver nanoparticles was calculated as 95.3% at 72 h (Table 1). The degradation percentage was increased as increasing the exposure time of dye silver nanoparticles complex in sunlight (Figure 10). Absorption peak for methylene blue dye was cantered at 660 nm in visible region which diminished and finally it disappeared while increasing the reaction time, which indicates that the dye had been degraded.

## 4. Conclusion

Green nanotechnology is gaining importance due to the elimination of harmful reagents and provides effective synthesis of expected products in an economical manner. Green synthesis of silver nanoparticles shows more compatible, ecofriendly, low cost, and less time consuming process. Herein, the silver nanoparticles were synthesized by using plant leaf extract of* M. tinctoria *under different pH. Silver nanoparticles formation was not detected in the acidic medium. In the alkaline medium, the size as well as the quantity of the silver nanoparticles formed is strongly dependent on the pH characterized by UV-Vis spectrophotometer. Spherical shape of the nanoparticles with the size ranges from 79 to 96 nm was confirmed by SEM. Crystalline nature was characterized by XRD, and presence of elemental silver was analyzed by EDX spectrum. The photocatalytic activity of green synthesized silver nanoparticles was evaluated by choosing methylene blue dye. The main absorption peak at 660 nm decreased gradually with the extension of the exposure time indicating the photocatalytic degradation of methylene blue dye. The present study, it is found that the use of natural renewable and eco-friendly reducing agent used for synthesis of silver nanoparticles exhibits excellent photocatalytic activity against dye molecules and can be used in water purification systems and dye effluent treatment.

## Figures and Tables

**Table 1:** Methylene blue degradation (%) by synthesized silver nanopartilces (10 mg) analyzed by triplicate experiments.

Exposure time	Amount of degradation of dye (%)
1 h	1.1 ± 0.30
2 h	4.7 ± 0.15
3 h	6.3 ± 0.25
4 h	10.5 ± 0.47
21 h	15.3 ± 0.14
22 h	19.5 ± 0.65
24 h	29.5 ± 0.33
41 h	32.6 ± 0.20
42 h	44.2 ± 0.10
44 h	47.9 ± 0.50
45 h	53.2 ± 0.75
46 h	54.7 ± 0.27
48 h	58.9 ± 0.18
65 h	63.2 ± 0.23
66 h	67.6 ± 0.58
70 h	75.8 ± 0.67
71 h	82.1 ± 0.15
72 h	95.3 ± 0.30

±Standard deviation.
